# The Beijing angle closure progression study: design and methodology

**DOI:** 10.3389/fmed.2024.1385060

**Published:** 2024-07-17

**Authors:** Zhi-qiao Liang, Kang-yi Yang, Kun Lv, Yao Ma, Cun Sun, Ge Liang, Yan-kun Yue, Jia-yin Qin, Yao Zhao, Jia-nan Zhang, Qiong Yi, Xing-zhi Sun, Hui-juan Wu

**Affiliations:** ^1^Beijing Key Laboratory of Diagnosis and Therapy of Retinal and Choroid Diseases, Department of Ophthalmology, Peking University People’s Hospital, Eye Diseases and Optometry Institute, Beijing, China; ^2^College of Optometry, Peking University Health Science Center, Beijing, China; ^3^Department of Ophthalmology, Beijing Hui People's Hospital, Beijing, China; ^4^Department of Ophthalmology, The PLA Rocket Force Characteristic Medical Center, Beijing, China; ^5^Department of Ophthalmology, Capital Medical University Fuxing Hospital, Beijing, China; ^6^Department of Ophthalmology, Peking University International Hospital, Beijing, China; ^7^Department of Ophthalmology, Beijing Zhanlanlu Hospital, Beijing, China; ^8^Department of Ophthalmology, Beijing Nuclear Industry Hospital, Beijing, China; ^9^Department of Ophthalmology, Beijing Hepingli Hospital, Beijing, China; ^10^Ping An Healthcare Technology, Beijing, China

**Keywords:** progression of angle closure, primary angle-closure suspect, primary angle-closure, primary angle-closure glaucoma, incidence, risk factors

## Abstract

**Purpose:**

The purpose of this study is to summarize the design and methodology of a large-scale trial in northern China, the Beijing Angle Closure Progression Study (BAPS). This trial is designed to explore the 5-year incidence of primary angle-closure suspect (PACS) progressing to primary angle-closure (PAC) or primary angle-closure glaucoma (PACG) and to determine the possible risk factors of disease progression.

**Methods/design:**

The BAPS is a clinic-based, multicenter, noninterventional trial conducted on a sample of urban Chinese adults. Consecutive eligible patients who meet PACS diagnostic criteria will be recruited from eight participating centers, with the trial commencing on August 4, 2022. The target sample size is set at 825 subjects, with follow up planned for a minimum period of 5 years. Baseline examination will include presenting visual acuity, best corrected visual acuity, intraocular pressure (IOP), undilated slit-lamp biomicroscopy, stereoscopic evaluation of the optic disc, visual field test, optical coherence tomography evaluation of retinal nerve fiber layer, ultrasound biomicroscopy and IOLMaster. Questionnaires will also be used to collect detailed personal history. Patients are scheduled to visit the glaucoma clinic every 12 months and may visit the emergency room in case of acute attack of angle closure. Study endpoints include acute PAC episodes, elevated IOP, peripheral anterior synechiae, glaucomatous visual field defect, or glaucomatous abnormality of optic nerve.

**Discussion:**

The BAPS will provide data on the 5-year incidence of PACS progressing to PAC or PACG and determine the risk factors for disease progression. This study will also help redefine high-risk patients with PACS.

## Introduction

Although the global prevalence of primary angle-closure glaucoma (PACG) is less than half that of primary open-angle glaucoma, it poses a significantly higher risk of causing severe bilateral blindness, with rate three times greater than primary open-angle glaucoma (1). PACG is a leading cause of irreversible blindness worldwide (2), characterized by the closure of the anterior chamber angle. Primary angle-closure suspect (PACS), the earliest stage of primary angle-closure diseases (PACD), is defined as 6 or more clock hours of appositional contact between the peripheral iris and posterior trabecular meshwork on gonioscopy. Progression from PACS to primary angle-closure (PAC) involves elevated intraocular pressure (IOP) or the presence of peripheral anterior synechiae (PAS), while PACG involves these features along with glaucomatous optic neuropathy or glaucomatous visual field defects (3). It is estimated that by 2040, the number of PACG cases worldwide is projected to reach 32 million, with a significant proportion occurring in Asia (4, 5). Therefore, a better understanding of the natural history of PACD may play an important role in preventing devastating visual impairment.

Previous studies have reported the prevalence of PAC and PACG ranging from 1 to 11.3% (6–9). However, longitudinal data on the incidence and progression of earlier stages of angle closure that precede PAC/PACG are scarce, particularly in Asian population (10–14). For instance, a Danish study demonstrated that the rate of progression from the condition of shallow anterior chamber to PACG was 16% over 10 years (15). In Asia, PACS progression rates to PAC or PACG range from 5.3 to 25.5% (10–14). Identified risk factors for progression include bilateral PACS (10), smaller angle width (11), shorter angle open distance, flatter iris curvature, and older age (12).

Most studies assessing anterior chamber characteristics have utilized examinations like IOLMaster, A-scan, and anterior segment-optical coherence tomography (AS-OCT). However, the ultrasound biomicroscopy (UBM) has not been widely used, despite its valuable role in evaluating angle closure. UBM can provide detailed information on the volume of the ciliary body, the degree of ciliary anterior rotation, and parameters related to the vitreous zonule (VZ), which are often overlooked (16). Historically, laser peripheral iridotomy (LPI) was considered to be a precaution against progression of PACD, but recent studies postulated that the benefit of prophylactic LPI was limited for PACS (17). Therefore, identifying PACS patients with high progression risks accurately is imperative to apply LPI selectively.

The Beijing Angle Closure Progression Study (BAPS) aims to explore the 5-year incidence of PACS progressing to PAC or PACG and identify potential risk factors for disease progression. This study seeks to provide evidence for developing accurate management strategies for PACS.

## Materials and methods

### Study design and principal aims

The BAPS is a clinic-based, multicenter, noninterventional trial conducted on a sample of urban Chinese adults. This study has been approved by the ethics committee of Peking University People’s Hospital and will adhere to the tenets of the Declaration of Helsinki. The protocol is prospectively registered at www.cilinicaltrials.gov (registration number NCT05563623). Institutional Review Board/Ethics Committee approval has been obtained.

The comprehensive study protocol is summarized in Table 1, providing an overview of the study design and key steps.

**Table 1:** Comprehensive study protocol for the Beijing angle closure progression study.

Step	Description	Details
Study design	Clinic-based multicenter noninterventional trial	Conducted on a sample of urban Chinese adults
Ethics approval	Approved by the Ethics Committee	Peking University People’s Hospital, follows the Declaration of Helsinki
Registration	Protocol registered	www.clinicaltrials.gov (NCT05563623)
Participant recruitment	Recruitment centers	8 participating centers
Inclusion criteria	Criteria for eligibility	6 or more clock hours of angle circumference with no PAS and normal IOP Aged 40–75 years Provide informed consent
Exclusion criteria	Criteria for exclusion	Evidence of PAC or PACG Previous intraocular surgery or laser treatment Severe health problems
Baseline examination	Comprehensive initial examination	Visual acuity IOP measurement Slit-lamp biomicroscopy Gonioscopy OCT UBM
Follow-up visits	Regular follow-up schedule	Every 12 months, including examinations for IOP, visual acuity, gonioscopy, UBM, OCT, and visual field testing
Study endpoints	Criteria for study endpoint	Acute PAC episode Elevated IOP (>21 mmHg) Presence of PAS Glaucomatous visual field defects
Data management	Handling and storing data	Non-image data in BAPS Database Image/original data stored securely Privacy of participants protected
Statistical analysis	Data analysis methods	SPSS 22.0 for analysis Significance level: 0.05 Independent *t*-test, Mann–Whitney U test, Pearson chi-square test, logistic regression analysis, Cox regression analysis

IOP, intraocular pressure; PAS, peripheral anterior synechiae; PAC, primary angle-closure; PACG, primary angle-closure glaucoma; OCT, optical coherence tomography; UBM, ultrasound biomicroscopy; BAPS, Beijing angle closure progression study.

**Figure 1:**
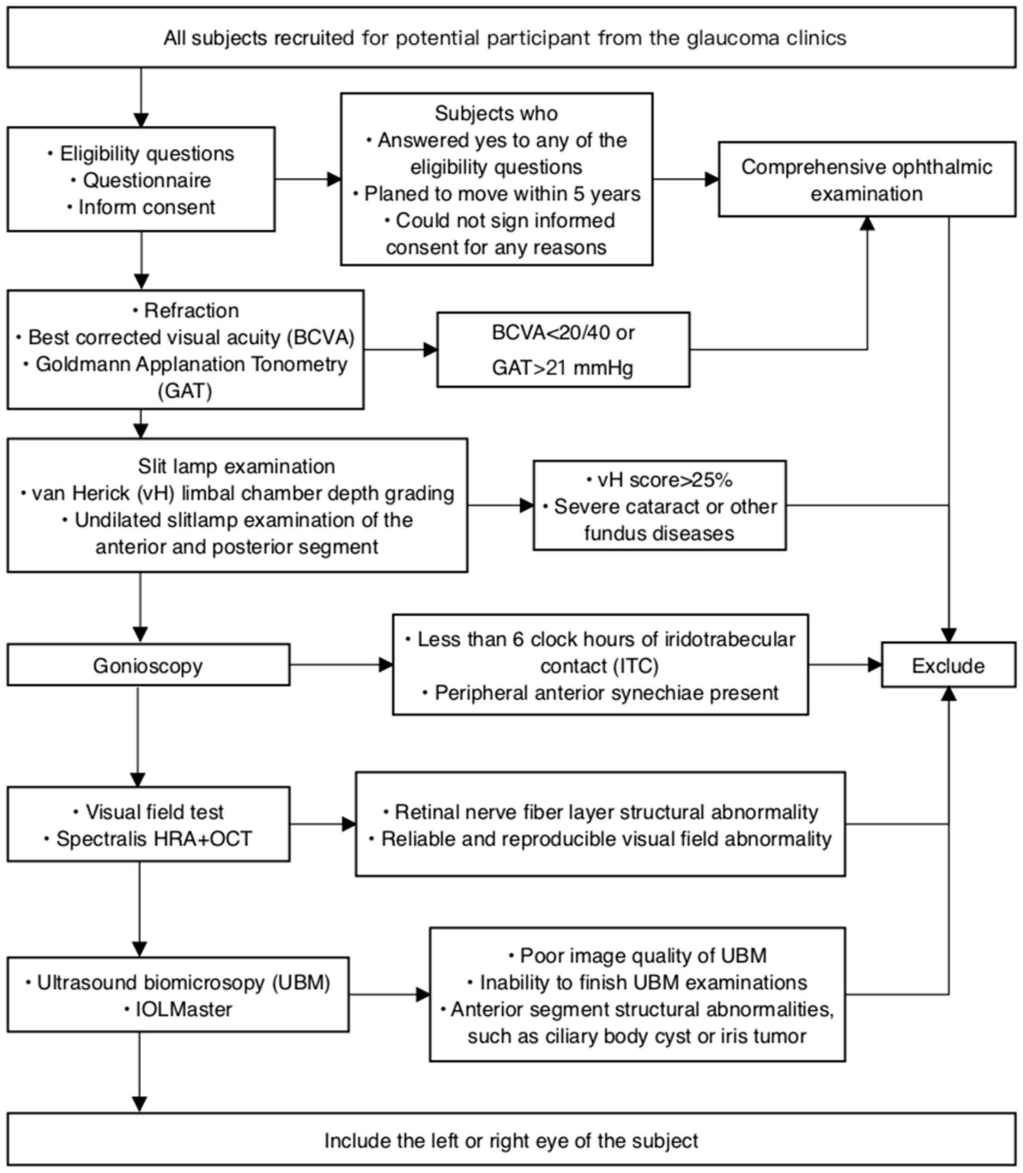
Outline of the workflow for screening and enrollment in the BAPS.

Consecutive eligible patients will be recruited from 8 participating centers, with the trial commencing on August 4th, 2022. Patients who agree to participate will undergo a comprehensive baseline examination, while those who decline will be asked to provide a reason. Both verbal and written informed consent will be obtained from every patient (Figure 1).

The principal aim of this trial is to explore the 5-year incidence of PACS progressing to PAC or PACG and to determine the risk factors for disease progression.

### Sample size estimation

Currently, many studies have reported the prevalence of PACS in the population, with rates ranging from 4.68% (95% Confidence Interval [CI]: 4.541–4.819%) to 10.4% (95% CI: 9.6–11.2%) (7–9, 18–21). However, few studies have investigated the incidence of PACS progressing to PAC or PACG (11, 12, 15, 22, 23). In a population-based prospective study conducted in India, 11 out of 50 PACS eyes (22%; 95% CI: 9.8–34.2%) developed PAC over 5 years (23). Another follow-up study in Denmark involving 75 PACS eyes reported a progression incidence of 16% (15). Given the similarity between our sample characteristics and those in the Indian study, we conservatively estimated the incidence of PACS progressing to PAC or PACG at 20%. To analyze the risk factors, we plan to collect a larger sample size than previous studies. Considering the number of candidate risk factors and the prevalence of PACS, we will use standard sample size calculation formulas for proportions, aiming for a desired level of statistical power of 80% and a significance level of 0.05. Based on these calculations, we estimate that 700 subjects are needed to achieve reliable results. Accounting for an anticipated attrition rate of up to 15%, we set the final sample size at 825 individuals, expecting to have complete follow-up data for 700 individuals at the end of the study.

### Clinical data collection

All subjects will be screened through a preliminary survey to verify their eligibility for recruitment in this trial. A comprehensive baseline examination will be conducted for every enrolled subject. Patients will be monitored for 5 years. All examinations involving subjective judgment and manipulation, such as Goldmann applanation (GAT), UBM, and gonioscopy, will be performed by experienced examiners.

For the analysis, we will enroll only one eye per subject to avoid correlation issues between eyes of the same individual. The selection of which eye to include will be randomized to ensure that there is no selection bias.

Inclusion criteria are as follows: (1) Static gonioscopy identifying six or more clock hours of angle circumference in which the posterior (usually pigmented) trabecular meshwork is not visible in both eyes with no PAS, and normal IOP, optic nerve, and visual field; (2) Patients aged between 40 and 75 years; and (3) Patients capable of providing informed consent.

Exclusion criteria are as follows: (1) Any evidence of PAC, characterized by a narrow angle as defined above, but with PAS and/or IOP > 21 mmHg, or PACG, indicated by a visual field defect or glaucomatous optic neuropathy; (2) Previous intraocular surgery or laser treatment, including cataract surgery, laser trabeculoplasty, trabeculectomy, laser peripheral iridectomy, and laser iridoplasty; (3) Signs of prior acute attack, such as glaucomatous fleck, keratic precipitates, or iris atrophy; (4) Anterior segment structural abnormalities, such as iris or ciliary body tumor; (5) Severe health problems that preclude follow-up, such as end-stage heart disease, kidney disease, lung disease, or terminal cancer; (6) Severe eye diseases requiring treatment, such as cataract, macular disease, and retinal detachment; and (7) Patients who plan to move from the area within the next 5 years.

### Screening survey

#### Eligibility questions

To expeditiously exclude individuals not qualified to participate in this trial, five screening questions will be asked. Participant who respond affirmatively to any of the following questions will be excluded: (1) Do you plan to relocate from this area within the next 5 years? (2) Have you undergone previous intraocular ophthalmic surgery? (3) Have you ever experienced serious eye trauma? (4) Have you been previously treated for glaucoma? (5) Do you have a severe, life-threatening disease, such as end-stage cardiac, renal, or pulmonary disease, or terminal cancer?

#### Informed consent

Ophthalmologists will elucidate the procedures and purpose of this study to potential participants. Should participants or their relatives raise any questions, the physicians will provide detail answers. Informed consent will be obtained from participants only after they have demonstrated a comprehensive understanding of the study.

#### Questionnaire

We will condense the information potentially associated with the onset of PACG into a more concise questionnaire. The questionnaire will include the following components: (1) Personal history, covering education level, employment status (retired or not), daily exercise duration, smoking and alcohol consumption history, family history of glaucoma, light exposure environment (self-reported indoor and outdoor time), and self-assessed personality traits; (2) Detailed ocular history, including ocular surgeries, laser treatment history, and history of ocular trauma; and (3) General medical history, including hypertension, diabetes, coronary heart disease and relevant conditions.

### Ophthalmologic examinations

All participants will undergo a comprehensive ocular examination, including presenting visual acuity (PVA), best-corrected visual acuity (BCVA), and IOP measurement with GAT (Haag-Streit, Koniz, Switzerland). IOP will be measured three times, and the average value recorded. Additional examinations will include undilated slit-lamp biomicroscopy and stereoscopic evaluation of the optic disc using a 90-diopter lens (Volk Optical, Inc., Mentor, OH). PVA and BCVA will be converted to logarithm of the minimal angle of resolution (LogMAR) scores from Snellen acuity. In a safe manner, lens grading will be assessed using a modified version of the Lens Opacity Classification System III (LOCS III) grading system. This assessment will take place subsequent to pupil dilation. We have included the evaluation of IOP and anterior chamber morphology before and after pupil dilation in our study protocol. Limbal anterior chamber depth (LACD) will be evaluated according to the modified van Herick system as a percentage of the adjacent corneal thickness. Patients with LACD ≤25% of peripheral temporal corneal thickness will be referred to gonioscopy. Gonioscopy will be performed in a dimly lit room [illumination less than 10 lux, measured with a luminance meter (Model ST-92, Beijing Teachers University Photoelectricity Instrument Factory, Beijing, China)] by a glaucoma specialist using a Zeiss-style four-mirror gonioscopy lens (Model G-4, Volk Optical, Inc., Mentor, OH) at 16× magnification, both with and without indentation. Five measurements of axial length (AL) will be taken using the IOLMaster 700 (Carl Zeiss Meditec, Inc., Dublin, CA) with a signal-to-noise ratio of more than 100, and the mean value will be used for analysis. Ocular biometry obtained from IOLMaster will include central cornea thickness, anterior chamber depth (ACD), lens thickness (LT), AL, flat keratometry (flat K), steep keratometry (steep K), and white-to-white (WTW) distance. AS-OCT (RTVue 100-2, Optovue, Fremont, California) will be employed to assess anterior segment structures and parameters such as corneal thickness, ACD, angle width, and iris curvature, in a non-contact, and high-resolution manner.

Optical coherence tomography (Spectralis HRA + OCT, Heidelberg Engineering GmbH, Heidelberg, Germany) will be employed to assess retinal nerve fiber layer (RNFL) defects. The visual field (VF) will be evaluated using the Swedish Interactive Threshold Algorithm (SITA) program 24-2 on a Humphrey Field Analyzer (Carl Zeiss Meditec, Inc., Dublin, CA) to identify characteristic glaucomatous VF defect. A characteristic glaucomatous defect is defined as a visual field abnormality consistent with the pattern of optic nerve damage caused by glaucoma (3).

UBM (Aviso, Quantel Medical, Inc., Bozeman, MT) measurements will be conducted using a 50-MHz transducer by well-trained operators blind to the clinical data. All patients will undergo UBM imaging in a supine position under room light [illumination 120 lux, measured with a luminance meter (Model ST-92, Beijing Teachers University Photoelectricity Instrument Factory, Beijing, China)]. After topical anesthesia with oxybuprocaine eye drops, an eyecup containing normal saline will be carefully mounted on the globe. The UBM transducer will be moved perpendicularly over the ocular structures without exerting pressure on the globe or touching the cornea. Measurements will be taken from both eyes in the superior, inferior, temporal, and nasal quadrants, as well as nasal-temporal scans centered on the pupil to obtain a full view of the anterior segment. During dynamic scanning, if the VZ appears on the monitor, the image will be stored. The strand attaching to the posterior VZ insertion zone and posterior lens equator, referred to as PVZ INS-LE, will also be categorized as VZ. All images will be acquired under consistent room lighting condition. Only images with a clear view of the scleral spur, angle, ciliary body, iris, and anterior surface of the lens will be included in the analysis.

We will use the built-in caliper to measure UBM parameters quantitatively in all four quadrants. Two examiners (KL and ZQL) who are blind to the clinical data will perform the measurements. The SS is a key landmark for the curvature of the inner angle wall, appearing as a protrusion of the sclera inward. We will measure the following parameters on full view scans at the nasal-temporal position: (1) Lens vault: the vertical distance from the anterior pole of the lens to the line connecting the two SSs, (2) Anterior chamber width: the distance between the two SSs (24), and (3) ACD: the axial distance from the corneal endothelium to the anterior lens surface (25). On radial scans at the superior, nasal, inferior, and temporal positions, we will measure these parameters: (1) Ciliary process area: the cross-sectional area of ciliary process enclosed by a line from the iris insertion to the ciliary body and a line from the point 500 μm from the SS perpendicular to the inner scleral wall to the ciliary process, and by the ciliary process surface internally (26), (2) Trabecular–ciliary angle: the angle between the posterior corneal surface and the anterior ciliary body surface, (3) Trabecular-ciliary process distance: a line from 500 μm anterior to the SS on the corneal endothelium toward the ciliary processes, (4) Angle opening distance at 500 μm and 750 μm: the distance from the posterior corneal surface to the anterior iris surface perpendicular to the trabecular meshwork at 500 μm and 750 μm from the SS (27), (5) Trabecular-iris angle: the angle between a line from 500 μm and 750 μm from the SS on the trabecular meshwork and a line from the SS to the opposite iris point, (6) Trabecular–iris space area at 500 μm and at 750 μm: the area bounded by AOD 500 and AOD 750 in front, a line from the SS perpendicular to the inner scleral wall to the iris behind, the inner corneoscleral wall above, and the iris surface below (27), (7) Iris area: the area enclosed by the full length of the iris (from spur to pupil), and (8) VZ: zonular fibers that extend from zonular plexus in posterior pars plicata valleys to vitreous membrane near ora serrata (28).

We will assess the repeatability and reproducibility of our UBM parameters. The first examiner (KL) will repeat each measurement within 2 weeks to test intra-observer variability. The second examiner (ZQL) will independently measure on a different day to test inter-observer variability. The intra-class correlation coefficient will be used to calculate intra-observer and inter-observer variability.

### Follow-up visits

In the absence of special circumstances, such as and acute angle-closure attack, patients will visit the glaucoma clinic every 12 months as part of the BAPS. Follow-up examinations will include: BCVA, IOP, undilated slit-lamp biomicroscopy, gonioscopy, UBM, Spectralis HRA + OCT, and VF testing (if suspecting of glaucomatous damage).

### Study endpoint and termination of follow-up (censored data)

The study endpoint will be reached if the participant meets any of the following criteria: (1) An episode of acute PAC; (2) Any PAS; (3) IOP > 21 mmHg; (4) Any patchy TM pigmentation; and (5) Glaucomatous visual field defect, RNFL defects, or glaucomatous abnormality of optic nerve. Participants meeting at least one of the five criteria will be classified in the progression group. For patients experiencing an acute angle-closure attack, the time of progression will be recorded accurately. If chronic progression (PAS or glaucomatous abnormality of optic nerve) is detected during routine follow-up, the progression time will be recorded based on the follow-up time.

The following conditions will be considered reasons for termination of follow-up and will be defined as censored data: (1) Inability to continue follow-up for various reasons, such as severe systemic diseases or relocation; (2) Development of new severe ocular diseases requiring treatment, such as vision-threatened cataract, retinal detachment, or age-related macular degeneration; (3) Missing 3 out of 6 monthly follow-up visits; and (4) Requesting withdrawal of informed consent.

### Data management

Two types of data will be generated in the BAPS: (1) non-image data, which will be entered into the BAPS Database according to information recorded in the written data forms during the trial procedure; and (2) image or other forms of original data obtained from the examination instruments. The parameters of UBM will be measured by the same investigator (KL). A portable hard disc of Universal Serial Bus (USB) will keep securely locked in cabinets at the research clinic established at the trial site for data storage, with duplicate copies backed up weekly. Participants’ privacy will be protected throughout the trial, ensuring confidentiality at all stages.

### Statistical analysis plan

All parameters measured in UBM will be calculated by averaging the corresponding measurements from both horizontal and vertical images. The normality of the data will be evaluated by using the Shapiro–Wilk test. Means and standard deviations will be calculated for all continuous variables. Baseline characteristics will be compared between progression group and non-progression group using the independent *t*-test or Mann–Whitney U test for continuous variables and the Pearson chi-square test for categorical variables.

The primary outcome is the progression rate, in other words, the rate of acute PAC (IOP > 21 mmHg) and the proportion of eyes developing PAS. We will evaluate the risk factors for PACS progression to PAC or PACG using univariable and multivariable logistic regression analysis, as well as Cox regression analysis. All statistical analyses will be performed using the software SPSS 22.0 (SPSS, Inc., Chicago, IL) with a significance level set at 0.05.

## Discussion

The BAPS is a multicenter, randomized, observational trial designed to explore the 5-year incidence of PACS progressing to PAC or PACG and to determine the risk factors for disease progression. The study hypothesizes that a subset of PACS patients, despite having similar initial presentations, might be predisposed to a faster disease progression, while others may remain relatively stable over time. By thoroughly analyzing and understanding the variables contributing to this divergence in disease course, we aim to identify high-risk PACS individuals who are more likely to developing PAC or PACG. This could enable better clinical decisions regarding whether to opt for prophylactic treatment or continued observation for these high-risk patients.

Although awareness of glaucoma screening is gradually increasing, the prevalence of PAC and PACG is as high as 1% in China (5, 6). Since the mid-1970s, LPI has been considered the first-line therapy for PAC and PACG (29). However, several randomized trials of screening and prophylactic LPI in patients with PACS conducted in Asia have reported no benefit in preventing vision loss due to glaucoma or in slowing the progression of the disease (17, 30). An Indian study demonstrated that LPI accelerates the progression of cataracts over a 6-years follow-up (31). Additionally, LPI may cause structural zonular damage and occult lens subluxation (32). Despite eye care providers being aware of these potential complications, a large number of LPI are still performed due to the fear of PACS rapidly evolving into PAC or PACG (33, 34). Therefore, identifying patients with high-risk PACS is critical both for reducing complications from LPI and for mitigating damage to visual function from PAC and PACG.

Alsbirk et al. conducted a study on angle closure in the population of Greenland Eskimos, who exhibited a high incidence of PACG, similar to conditions observed in Southeast Asian populations (15). The study reported that at a 10-year follow-up, 16% (95% CI, 8.5–26.3%) of 75 eyes with PACS progressed to the equivalent of ISGEO PAC (synechial PAC) or PACG (acute or chronic). Slit-lamp screening (LACD and ACD) was effective in identifying a subgroup at risk for developing PACG over the 10-year follow-up (15). However, the study has two significant limitations. First, the use of slit lamp alone to assess LACD and ACD is subjective and unreliable. Second, the study did not use the ISGEO classification, as it was conducted earlier and applied previous diagnostic criteria (35). Consequently, it cannot provide valuable guidance for the treatment of PACD. Wishart and Batterbury’s study reported that 36% of patients in the narrow angle group developed PACG over an average 4-year follow-up period (36). This study faced similar issues as Alsbirk et al.’s study, and it was not observational, as it employed IOP-lowering medications for patients. Therefore, it could not provide valuable insights into the natural history of PACD.

An Indian prospective study demonstrated that 22% (95% CI, 9.8–34.2%) of patients with PACS developed PAC, but no PACG were observed during the 5-year follow-up. The study identified bilateral PACS as a clinical risk for disease progression, but no biometric characteristics were found to be significant (10). The sample size in this study was relatively small, with only 50 PACS were examined, and the participants were not consecutively enrolled. Instead, the patients previously diagnosed as PACS in other studies were invited for a review examination. Consequently, the incidence of disease progression may be biased. Additionally, the absence of PACS cases progressing to PACG over the 5-years period is inconsistent with clinical experience.

Several other population-based studies conducted in China (11, 12) and Singapore (13) share similar aims and designs with this study. They have numerous advantages, such as being multicenter, having large sample size, offering valuable clinical significance, employing scientific study design, and incorporating long follow-up periods. However, none of these studies included UBM in their baseline examinations. Anterior segment imaging techniques, including AS-OCT and UBM, has advantages over gonioscopy (37), such as providing more detailed parameters of the iris (26), lens (38), and anterior chamber (39). Increasing evidence suggests that structures behind iris, such as the ciliary body (26, 40) and VZ (41), play important roles in the pathogenesis of PACD. UBM is the only technique capable of providing sufficient details of these posterior structures. To the best of our knowledge, this study is the first to explore the natural course of PACD including UBM data.

## Conclusion

The BAPS is a large-scale, multicenter, clinic-based trial conducted in an urban setting. The study aims to provide valuable data on the 5-year incidence of PACS progressing to PAC or PACG and to determine the risk factors for disease progression. Additionally, this study may help redefine high-risk patients with PACS and offer guidance on assessing the benefits and risks of PACS treatment.

## Ethics statement

This study has been approved by the Ethics Committee of Peking University People’s Hospital and follows the tenets of the Declaration of Helsinki. The patients/participants provided their written informed consent to participate in this study.

## Author contributions

Z-qL: Formal analysis, Methodology, Writing – original draft, Writing – review & editing. K-yY: Data curation, Formal analysis, Validation, Writing – original draft. KL: Data curation, Formal analysis, Validation, Writing – original draft. YM: Formal analysis, Investigation, Writing – original draft. CS: Data curation, Methodology, Supervision, Writing – original draft. GL: Data curation, Methodology, Supervision, Writing – original draft. Y-kY: Data curation, Methodology, Supervision, Writing – original draft. J-yQ: Data curation, Methodology, Supervision, Writing – original draft. YZ: Data curation, Methodology, Supervision, Writing – original draft. J-nZ: Data curation, Methodology, Supervision, Writing – original draft. QY: Data curation, Methodology, Supervision, Writing – original draft. X-zS: Formal analysis, Writing – original draft. H-jW: Data curation, Formal analysis, Funding acquisition, Investigation, Methodology, Supervision, Writing – original draft, Writing – review & editing.

## Glossary

BAPS: Beijing Angle Closure Progression Study
PACS: primary angle-closure suspect
PAC: primary angle-closure
PACG: primary angle-closure glaucoma
PACD: primary angle-closure diseases
IOP: intraocular pressure
PAS: peripheral anterior synechiae
LPI: laser peripheral iridotomy
UBM: ultrasound biomicroscopy
PVA: presenting visual acuity
BCVA: best corrected visual acuity
LogMAR: logarithm of the minimal angle of resolution
LOCS III: Lens Opacity Classification System III
LACD: limbal anterior chamber depth
AL: axial length
ACD: anterior chamber depth
LT: lens thickness
WTW: white-to-white
AS-OCT: anterior segment-optical coherence tomography
RNFL: retinal nerve fiber layer
VF: visual field
SS: scleral spur
VZ: vitreous zonule
PVZ INS-LE: posterior vitreous zonule insertion zone and posterior lens equator
ISGEO: International Society of Geographical and Epidemiological Ophthalmology
